# Probiotics Supplementation Therapy for Pathological Neonatal Jaundice: A Systematic Review and Meta-Analysis

**DOI:** 10.3389/fphar.2017.00432

**Published:** 2017-06-30

**Authors:** Zhe Chen, Lingli Zhang, Linan Zeng, Xiaoyan Yang, Lucan Jiang, Ge Gui, Zuojie Zhang

**Affiliations:** ^1^Department of Pharmacy, West China Second University Hospital, Sichuan UniversityChengdu, China; ^2^Evidence-Based Pharmacy Center, West China Second University Hospital, Sichuan UniversityChengdu, China; ^3^Key Laboratory of Birth Defects and Related Diseases of Women and Children (Sichuan University), Ministry of EducationChengdu, China; ^4^Department of Neonatology, West China Second University Hospital, Sichuan UniversityChengdu, China; ^5^West China School of Pharmacy, Sichuan UniversityChengdu, China

**Keywords:** probiotics, neonatal jaundice, systematic review, meta-analysis

## Abstract

**Background:** Neonatal jaundice is a relatively prevalent disease and affects approximately 2.4–15% newborns. Probiotics supplementation therapy could assist to improve the recovery of neonatal jaundice, through enhancing immunity mainly by regulating bacterial colonies. However, there is limited evidence regarding the effect of probiotics on bilirubin level in neonates. Therefore, this study aims at systematically evaluating the efficacy and safety of probiotics supplement therapy for pathological neonatal jaundice.

**Methods:** Databases including PubMed, Embase, Cochrane Library, China National Knowledge Infrastructure (CNKI), Wan Fang Database (Wan Fang), Chinese Biomedical Literature Database (CBM), VIP Database for Chinese Technical Periodicals (VIP) were searched and the deadline is December 2016. Randomized controlled trials (RCTs) of probiotics supplementation for pathological neonatal jaundice in publications were extracted by two reviewers. The cochrane tool was applied to assessing the risk of bias of the trials. The extracted information of RCTs should include efficacy rate, serum total bilirubin level, time of jaundice fading, duration of phototherapy, duration of hospitalization, adverse reactions. The main outcomes of the trials were analyzed by Review Manager 5.3 software. The relative risks (RR) or mean difference (MD) with a 95% confidence interval (CI) was used to measure the effect.

**Results:** 13 RCTs involving 1067 neonatal with jaundice were included in the meta-analysis. Probiotics supplementation treatment showed efficacy [RR: 1.19, 95% CI (1.12, 1.26), *P* < 0.00001] in neonatal jaundice. It not only decreased the total serum bilirubin level after 3day [MD: −18.05, 95% CI (−25.51, −10.58), *P* < 0.00001], 5day [MD: -23.49, 95% CI (−32.80, −14.18), *P* < 0.00001], 7day [MD: −33.01, 95% CI (−37.31, −28.70), *P* < 0.00001] treatment, but also decreased time of jaundice fading [MD: −1.91, 95% CI (−2.06, −1.75), *P* < 0.00001], as well as the duration of phototherapy [MD: −0.64, 95% CI (−0.84, −0.44), *P* < 0.00001] and hospitalization [MD: −2.68, 95% CI (−3.18, −2.17), *P* < 0.00001], when compared with the control group. Additionally, no serious adverse reaction was reported.

**Conclusion:** This meta-analysis shows that probiotics supplementation therapy is an effective and safe treatment for pathological neonatal jaundice.

## Introduction

Neonatal jaundice is one of the most common conditions need medical care and affects approximately 2.4–15% of newborn babies during the first 2 weeks of life (Kelly and Stanton, 1995). About 60% of term and 80% of preterm infants develop jaundice in the first week of life and about 10% of breastfed babies are still jaundiced at 1 month (Xiong et al., 2012). The most common symptom in an infant with jaundice is the yellow discoloration of skin and sclera of neonates. It is caused by a raised level of bilirubin, a condition known as hyperbilirubinaemia (Rennie et al., 2010). The most common form of hyperbilirubinaemia observed in neonates is caused by unconjugated or unbound bilirubin (Carlo et al., 2015). Extreme elevation of unconjugated bilirubin can result in kernicterus, or even death.

Nowadays, probiotics attracts increased more and more attention, they are defined as live microorganisms, which may have health benefits for the host if consumed in adequate amounts (Harsharn and Jaya, 2008). The most commonly used probiotics are lactic acid bacteria and non-pathogenic yeasts (Eamonn, 2011). It has been demonstrated that probiotics could be applied to regulating the gastrointestinal disease, such as diarrhea, neonatal necrotizing enterocolitis, *Helicobacter pylori* eradication (Behnsen et al., 2013). Recently, probiotics and their products are well studied for their health benefit effects on immunomodulatory, type 2 diabetes, hypertension, neonatal jaundice and so on (Marta and Józef, 2015).

In recent years, probiotics has been used in the treatment of jaundice. Funda et al. suggested that Bifidobacterium species may protect against breast milk jaundice and probiotics may be protective against hyperbilirubinemia by affecting intestinal motility and intestinal microbial flora (Funda et al., 2013). Geyik et al. demonstrated that *Saccharomyces boulardii* has an effect on preventing bacterial translocation and improvement of intestinal barrier function in an obstructive jaundice animal model (Geyik et al., 2006). Claire et al. also showed the probiotic had an effect on obstructive jaundice through modulation of gut barrier function (Claire et al., 2013). The efficacy of probiotics depends upon their ability to pass through the stomach and duodenum, and colonize in the intestinal lumen, to reduce the overgrowth of bacteria in the small bowel, restore gastrointestinal barrier function and modulate the immune system (Eamonn, 2011).

The effect of probiotics in the treatment of neonatal jaundice is a new area for researchers and clinicians (Ozge et al., 2015). In clinical, besides from phototherapy, drugs, massage therapy, acupuncture treatment or exchange transfusion, probiotics supplementation therapy could assist to improve the recovery of neonatal jaundice, through enhancing immunity mainly by regulating bacterial colonies. They can form a biological barrier by specifically binding intestinal epithelial cells through teichoic acid (Ohland and Macnaughton, 2010). However, there is limited evidence regarding the effect of probiotics on bilirubin level in neonates. Therefore, this study aims at systematically evaluating the efficacy and safety of probiotics supplement for pathological neonatal jaundice.

## Methods

### Search strategy

We designed search strategies for three English language based electronic databases including PubMed, EMBase and Cochrane Library. Four Chinese electronic database including China National Knowledge Infrastructure (CNKI), WanFang Database (WanFang), Chinese Biomedical Literature Database (CBM), VIP Database for Chinese Technical Periodicals (VIP) were also searched. Other sources were used to find additional studies, such as Clinical Trials and The World Health Organization Clinical Trials Registry Platform, Cochrane Central Registry of Controlled Trials.

The following principal search terms and MESH headings were used: “probiotics” or “probiotic” or “Lactobacillus” or “Bifidobacterium” or “*S. boulardii*” or “yeast” or “yogurt” and “neonatal jaundice” or “neonatal hyperbilirubinaemia.” We would look for additional studies in reference lists of selected articles, contact with authors about knowledge of published or unpublished articles. The results of searches would be crosschecked in order to eliminate duplicates. The deadline of all retrieval was December 2016.

### Inclusion criteria

The following studies were included in analysis: (1) randomized controlled trials using clear and adequate randomization methods; (2) jaundice within 24 h after birth; (3) serum total bilirubin concentration increases more than 85.5 μmol/L (5 mg/dl) per day or more than 8.5 μmol/L per h; (4) studies published in either English or Chinese; (5) studies compared at least two treatment groups with (a) control patients underwent routine comprehensive treatment (includes phototherapy, enzyme inducer, immunoglobulin supportive treatment and so on) or phototherapy (b) patients received identical routine comprehensive treatment plus probiotics or identical phototherapy plus probiotics. (6) All studies were though the ethics review committee.

### Exclusion criteria

The following studies were excluded from the analysis: (1) Studies with incomplete or missing information; (2) Studies using inadequate or unclear randomization methods; (3) Suspected or documented neonatal infection such as chorioamnionitis, intrauterine infection, sepsis and urinary tract infection; (4) secondary jaundice caused by asphyxia, hemolytic disease, intracranial hemorrhage or other diseases.

### Data extraction

Data extraction form designed according to Cochrane Systematic Review Handbook (version 5.3) was used to extract the relevant information from each RCT independently. Two independent reviewers screened all the titles and abstracts to determine potential eligible articles. They independently and blindly applied the eligibility criteria to perform the final selection. When discrepancies occurred between both reviewers regarding the inclusion of the articles, they would discuss and identify the reasons to include or exclude the articles and then make the final decision. If they could not reach an agreement, the final decision would be based on a third reviewer.

### Risk of bias assessment

The Cochrane risk of bias tool was used to assess the risk of bias in RCT studies. The six domains of this tool included random sequence generation, allocation concealment, blinding of participants and personnel, blinding of outcome data, incomplete outcome data and selective reporting. The judgment was marked as “high risk,” “unclear risk,” or “low risk.” Trials that met all the criteria were categorized as low risk of bias, whereas those that met none were high risk of bias. The others were classified as unclear risk of bias if the information was insufficient to make a judgment.

### Data analysis

Meta-analysis was conducted with RevMan 5.3. The data from RCT studies was pooled and expressed as relative risks (RR) with 95% confidence interval (CI). Assessment of heterogeneity was done by I-squared (I^2^) statistics. A fixed effects model was initially conducted. If significant heterogeneity existed among trials (I^2^ > 50%), potential sources of heterogeneity was considered, and where appropriate a random effects model was used.

## Results

### Characteristics of the included studies

**Figure 1:**
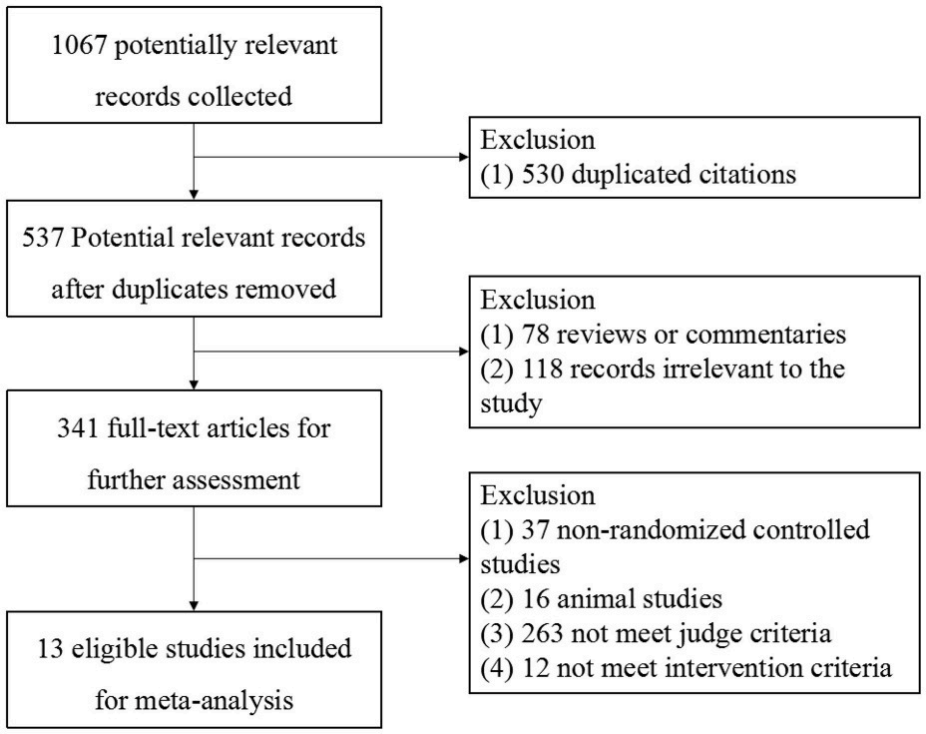
Flow diagram of selecting study.

A total of 1,067 records were identified for initial screening and 13 eligible articles were included in this meta-analysis (Figure 1). These studies were published between 2008 and 2016. There was no significant difference in ages, sex and disease course between two groups. Of these 13 articles, 5 were treated with combination of routine comprehensive treatment (RCT) and *Bifidobacterium*, 4 were treated with combination of RCT or phototherapy and *S. boulardii*, 1 were treated with combination of RCT and *Clostridium butyricum*, 1 was treated with combination of phototherapy and probiotic oligosaccharides, 2 were treated with combination of RCT and *Bacillus subtilis* (Table 1).

**Table 1:** Characteristics of included studies.

**References**	**Maternal age (weeks or days)**	**Weight (Kg)**	**Sex male/female**	Cases T/C^*^	**Interventions**		**Adverse reaction**
					**Treatment group**	**Control group**	
Zhang and Deng, 2008	T:37.4 ± 4.8	T:3.87 ± 0.25	T: 14/11	25/25	Bifidobacterium+RCT^*^	RCT^*^	No adverse reaction
	C:38.2 ± 3.3	C:2.75 ± 0.19	C: 13/12				
Liang, 2012	T:38.2 ± 0.5	NR^*^	T: 16/24	40/32	Bifidobacterium+RCT^*^	RCT^*^	6 cases of fever
	C:38.2 ± 0.4		C: 17/15				
Gamze et al., 2013	T:27.3 ± 5.6	T:1.15 ± 0.25	T: 45/36	81/98	Saccharomyces boulardii+phototherapy	Phototherapy	NR^*^
	C:27.8 ± 5.2	C:1.13 ± 0.28	C: 49/49				
Ozge et al., 2015	T:27.5 ± 5.3	T:3.07 ± 0.59	T: 26/32	58/61	Saccharomyces boulardii+phototherapy	Phototherapy	No adverse reaction
	C:27.2 ± 5.6	C:3.22 ± 0.61	C: 29/32				
He, 2014	T:5.1 ± 1.1	T:3.2 ± 0.9	NR^*^	42/42	Clostridium butyricum+RCT^*^	RCT^*^	6 cases of diarrhea, 4 cases of skin rash
	C:4.2 ± 1.3	C:3.2 ± 0.9					
Wang et al., 2014	T:7.3 ± 1.1	NR^*^	T: 29/27	56/50	Saccharomyces boulardii+RCT^*^	RCT^*^	No adverse reaction
	C:7.2 ± 1.3		C: 27/23				
Armanian et al., 2016	T:5.2 ± 2.0	T:1.26 ± 0.21	NR^*^	25/25	Probiotic oligosaccharides+ Phototherapy	Phototherapy	NR^*^
	C:4.2 ± 1.3	C:1.19 ± 0.19					
Liu et al., 2015	T:11.3 ± 1.2	T:3.86 ± 0.23	T: 22/12	34/34	Bifidobacterium +RCT^*^	RCT^*^	No adverse reaction
	C:11.2 ± 1.3	C:3.75 ± 0.21	C: 20/14				
Cen et al., 2015	T:3.72 ± 1.9	NR^*^	NR^*^	34/33	Bacillus subtilis+RCT^*^	RCT^*^	Fever, diarrhea, skin rash
	C:3.21 ± 2.0						
Xu et al., 2015	T:3.9 ± 1.2	NR^*^	T: 30/29	59/59	Saccharomyces boulardii+RCT^*^	RCT^*^	No adverse reaction
	C:4.1 ± 1.2		C: 32/27				
Zhang et al., 2015	T:15.6 ± 6.7	T:3.9 ± 1.7	T: 54/46	100/100	Bifidobacterium +RCT^*^	RCT^*^	3 cases of skin rash
	C:15.2 ± 6.6	C:3.6 ± 1.8	C: 56/44				
Lin, 2016	T:6.8 ± 2.1	NR^*^	T: 18/12	30/30	Bifidobacterium +RCT^*^	RCT^*^	1 case of fever and fatigue
	C:6.8 ± 2.2		C: 17/13				
Tian and Guo, 2016	T:4.1 ± 3.94	T:3.25 ± 0.79	T: 19/16	35/34	Bacillus subtilis+RCT^*^	RCT^*^	No adverse reaction
	C:4.2 ± 3.85	C:3.36 ± 0.83	C: 18/16				

* *T, Treatment group; C, Control group*.

* *NR, Not reported*.

* *RCT, Routine comprehensive treatment (includes phototherapy, enzyme inducer, immunoglobulin supportive treatment and so on)*.

### Methodological quality of included trials

**Figure 2:**
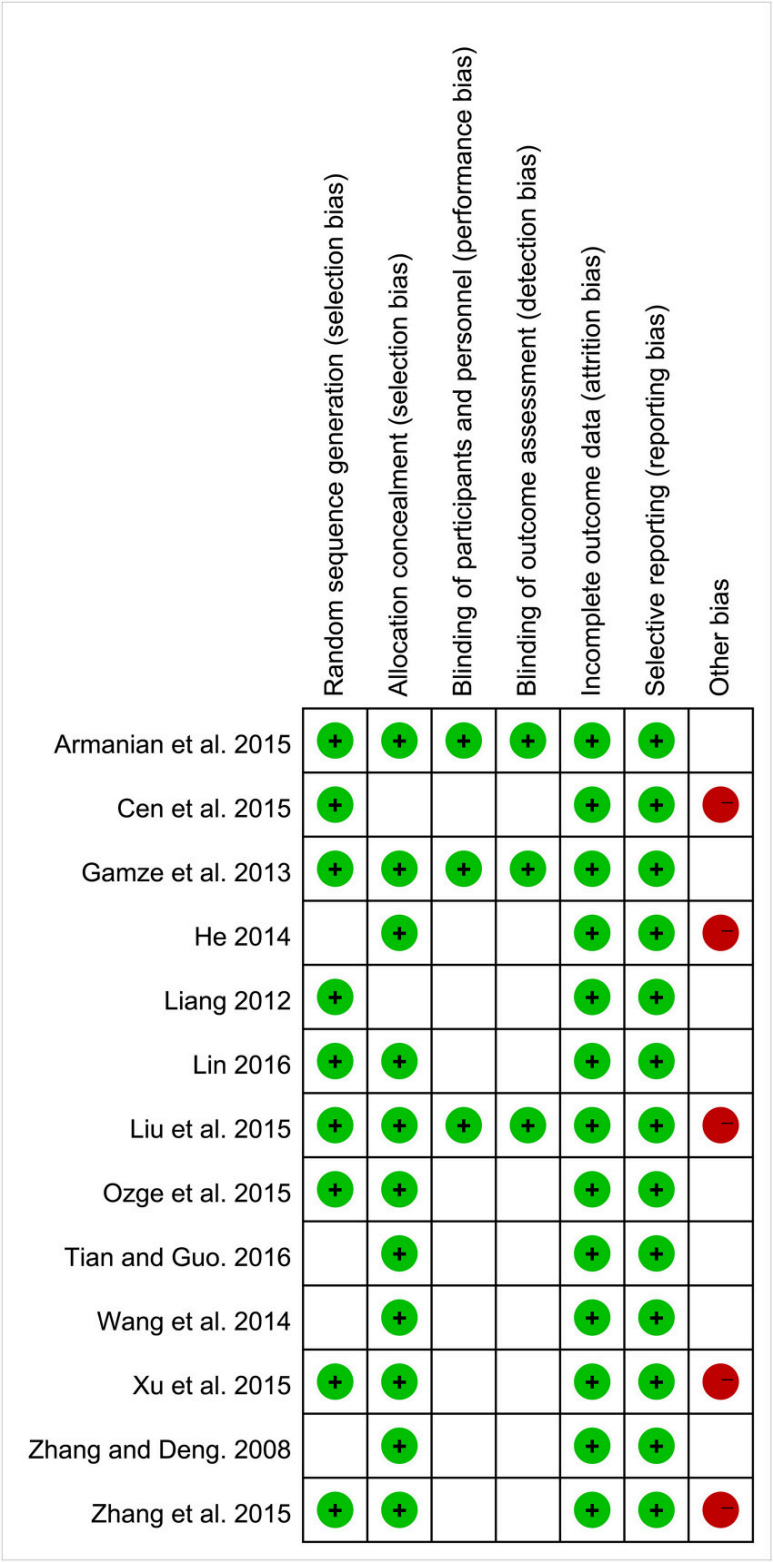
Methodological quality assessment for risk of bias for each included study.

According to Cochrane risk of bias estimation, all trials were mentioned random. But only 9 trials referred to the specific method of random. Eleven trials performed on allocation concealment. Three trials performed blinding of participants, personnel assessment, and blinding of outcome assessment. All the trials reported on complete outcome data and selective reporting. Eight trials were low risk in other bias (Figure 2).

### Primary outcomes

#### Efficacy rate

**Figure 3:**
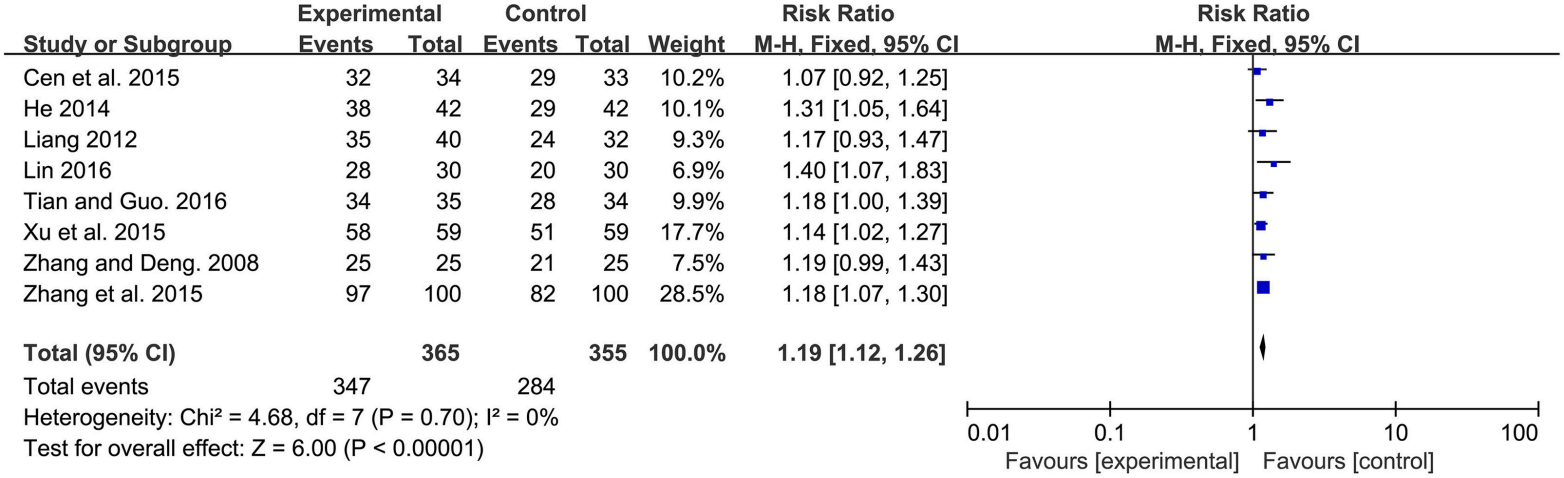
Forest plot of efficacy rate.

Among the 13 studies, 8 studies contributed to this analysis. There was significant improvement of efficacy rate in experimental group for neonatal jaundice [RR: 1.19, 95% CI (1.12, 1.26), *P* < 0.00001]. There was no significant heterogeneity (*P* = 0.70, I^2^ = 0%) (Figure 3).

#### Serum total bilirubin level

**Figure 4:**
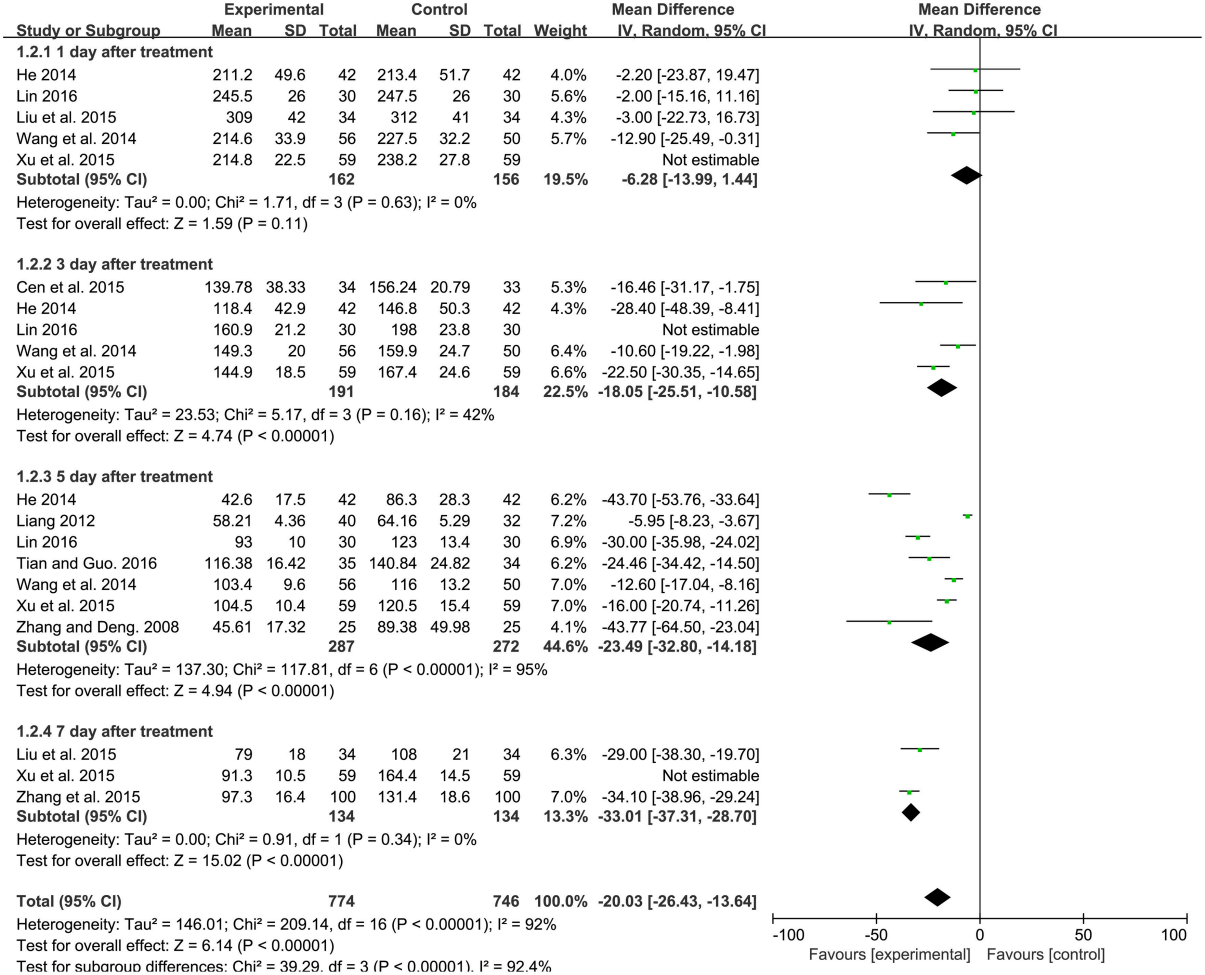
Forest plot of serum total bilirubin level.

Serum total bilirubin level, which was tested by quantitative method. Among the 13 studies, 10 studies contributed to this analysis. Before sensitivity analysis, the four subgroups had significant heterogeneity. After sensitivity analysis, there was no significant difference in decreasing bilirubin level between experimental group and control group after 1 day treatment [MD: −6.28, 95%CI (−13.99, 1.44), *P* = 0.11] with no heterogeneity. And there was a significantly lower bilirubin level in experimental group compared to the control group after 3 day [MD: −18.05, 95% CI (−25.51, −10.58), *P* < 0.00001], 5 day [MD: −23.49, 95% CI (−32.80, −14.18), *P* < 0.00001], 7 day [MD: −33.01, 95% CI (−37.31, −28.70), *P* < 0.00001] treatment for neonatal jaundice. Q-statistic indicated no heterogeneity was showed after 3 and 7 day treatment. However, heterogeneity was still high after 5 day treatment (*P* < 0.00001, I^2^ = 95%; Figure 4).

#### Time of jaundice fading

**Figure 5:**
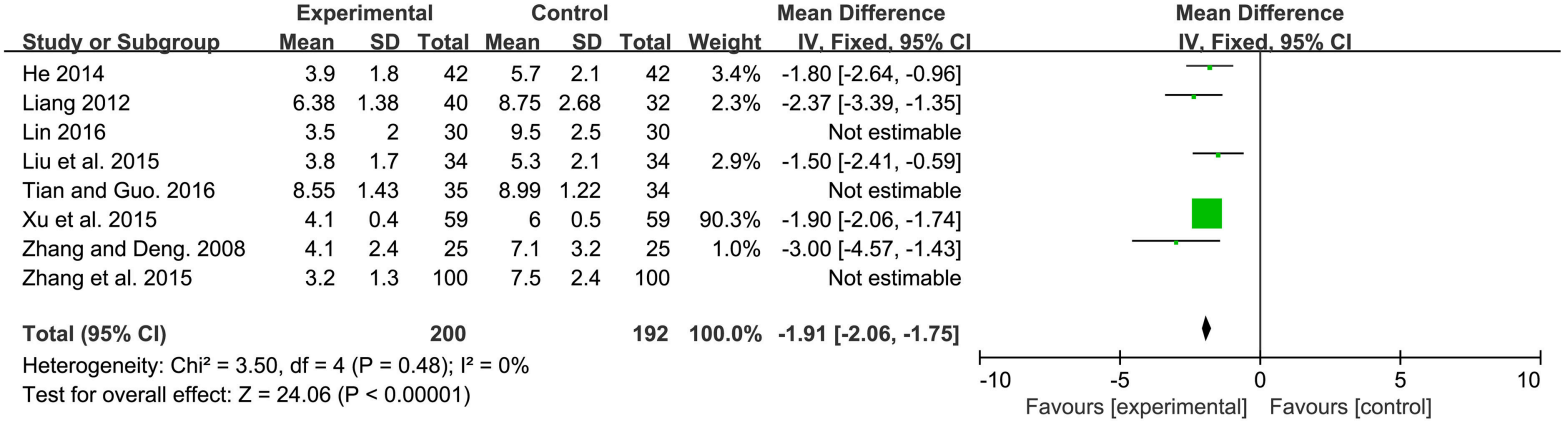
Forest plot of time of jaundice fading.

Time of defecation, defined as the duration from beginning of intervention to jaundice fading. Among the 13 studies, 8 studies contributed to this analysis. There was a significantly lower time of jaundice fading in probiotics supplementation group compared to the control group [MD: −2.62, 95% CI (−3.64, −1.61), *P* < 0.00001]. Heterogeneity was high (*P* < 0.00001, I^2^ = 95%). After sensitivity analysis, there was still a significantly lower time of jaundice fading in probiotics supplementation group compared to the control group [MD: −1.91, 95% CI (−2.06, −1.75), *P* < 0.00001] with no heterogeneity (*P* = 0.48, I^2^ = 0%; Figure 5).

### Secondary outcomes

#### Duration of phototherapy

**Figure 6:**
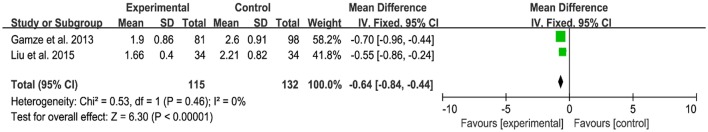
Forest plot of duration of phototherapy.

Only 2 of 13 RCT included in this analysis demonstrated a significant decrease in duration of phototherapy after administration of probiotics (Figure 6). And significant difference was found between the experimental group and control group [MD: −0.64, 95% CI (−0.84, −0.44), *P* < 0.00001]. There was no heterogeneity (*P* = 0.46, I^2^ = 0%).

#### Duration of hospitalization

**Figure 7:**
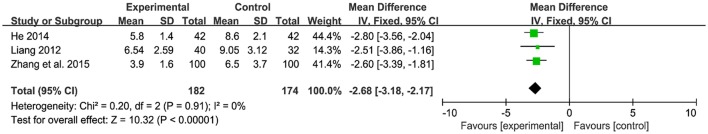
Forest plot of duration of hospitalization.

Only 3 of 13 RCT included in this analysis demonstrated a significant decrease in duration of hospitalization after administration of probiotics (Figure 7). And signification overall association was found between the experiment group and control group [MD: −2.68, 95% CI (−3.18, −2.17), *P* < 0.00001]. There was no heterogeneity (*P* = 0.91, I^2^ = 0%).

#### Adverse reactions

Among the 13 RCTs, 6 studies (Zhang and Deng, 2008; Wang et al., 2014; Liu et al., 2015; Ozge et al., 2015; Xu et al., 2015; Tian and Guo, 2016) reported no side effects. Five studies (Liang, 2012; He, 2014; Cen et al., 2015; Zhang et al., 2015; Lin, 2016) observed 20 cases of adverse reactions presented as fever, diarrhea, skin rash, fatigue. In spite of no difference between the treatment group and control group, further investigation was needed for a systematic safety assessment of probiotics supplementation therapy (Table 1).

## Discussion

Neonatal jaundice is a common pediatric disease in clinical, which can mainly be classified as physiological and pathological ones (Liu et al., 2015). The physiological jaundice is not an indication of an underlying disease, and this early jaundice is usually harmless. The pathological jaundice originates from various factors. If not treated, it easily leads to kernicterus or death. Pathological jaundice has many possible causes, including blood group incompatibility (most commonly ABO incompatibility), other causes of hemolysis (breaking down of red blood cells), sepsis (infection), liver disease and metabolic disorders. Deficiency of a particular enzyme, glucose-6-phosphate-dehydrogenase, can cause severe neonatal jaundice (NICE Guidance, 2010). This study mainly explored the effect of probiotics supplementation therapy for pathological jaundice in neonates.

This meta-analysis of 13 randomized controlled trials involving 1,067 subjects assessed the clinical value of probiotics supplementation therapy for the treatment of neonatal jaundice. It provided evidence that the combination of routine complementary treatment with probiotics supplementation therapy, including *Bifidobacterium, S. boulardii, C. butyricum*, probiotic oligosaccharides, *B. subtilis*, had an obvious increase of efficacy rate in neonatal jaundice. Moreover, it not only significantly improved neonatal jaundice by reducing total bilirubin, time of jaundice fading, but also decreased the duration of phototherapy and hospitalization. Among the 13 RCTs, 6 studies (Zhang and Deng, 2008; Wang et al., 2014; Liu et al., 2015; Ozge et al., 2015; Xu et al., 2015; Tian and Guo, 2016) reported no side effects. 5 studies (Liang, 2012; He, 2014; Cen et al., 2015; Zhang et al., 2015; Lin, 2016) observed 20 cases of adverse reactions presented as fever, diarrhea, skin rash, fatigue. In spite of no difference between the treatment group and control group, further investigation was needed for a systematic safety assessment of probiotics supplementation therapy (Table 1).

We also recognized that our study had limitations. First, because the value of jaundice fading in each guideline was different, the heterogeneity was high in time of jaundice fading. It suggested we should use the same guideline to detect the time of jaundice fading in future study. Second, according to Cochrane risk of bias estimation, randomized allocation of participants was mentioned in 9 trials. Most of the included studies only mentioned the use of random allocation, but they did not describe the specific random allocation method. So, it was hard for us to determine whether the allocation scheme was appropriate and whether blinding of participants and personnel was implemented. Some studies showed that unclear random allocation and allocation plan might exaggerate the hidden effect of up to 30–41% (Juni et al., 2001). Third, since RCTs of included studies centered in a short observation period and did not follow up the patients in long term, the methodological quality of clinical trials with probiotics supplementation therapy for neonatal jaundice needed further improvement. Therefore, well designed, large randomized, double blind, placebo-controlled trials would be required to further confirm the efficacy of probiotics. All of the outcome measures should be monitored by a standardized effective report system in clinical trials and rare serious adverse reaction could be observed through epidemiological studies.

## Conclusions

This systematic review and meta-analysis indicates that probiotics supplementation therapy is an effective and safe therapy option for the treatment of pathological neonatal jaundice with no serious adverse reaction. However, as the quality of included studies and the limitations of samples, the long-term efficacy and safety still need long-term and high-quality research confirmed.

## Author contributions

ZC analyzed the data and wrote the manuscript. LJ, GG, and ZZ collected and prepared samples. LZe and XY performed the analyses. LZh designed the study and amended the paper.

### Conflict of interest statement

The authors declare that the research was conducted in the absence of any commercial or financial relationships that could be construed as a potential conflict of interest. The reviewer XT and handling Editor declared their shared affiliation, and the handling Editor states that the process nevertheless met the standards of a fair and objective review.
